# Oligomerization status influences subcellular deposition and glycosylation of recombinant butyrylcholinesterase in *Nicotiana benthamiana*

**DOI:** 10.1111/pbi.12184

**Published:** 2014-03-11

**Authors:** Jeannine D Schneider, Sylvestre Marillonnet, Alexandra Castilho, Clemens Gruber, Stefan Werner, Lukas Mach, Victor Klimyuk, Tsafrir S Mor, Herta Steinkellner

**Affiliations:** 1 Department of Applied Genetics and Cell Biology, University of Natural Resources and Life SciencesVienna, Austria; 2 The Icon Genetics GmbHBio-Zentrum Halle, Germany; 3 Department of Chemistry, University of Natural Resources and Life SciencesVienna, Austria; 4 The Biodesign Institute, Arizona State UniversityTempe, Arizona, USA

**Keywords:** butyrylcholinesterase, sialic acid, glycoengineering, subcellular targeting, plants

## Abstract

Plants have a proven track record for the expression of biopharmaceutically interesting proteins. Importantly, plants and mammals share a highly conserved secretory pathway that allows similar folding, assembly and posttranslational modifications of proteins. Human butyrylcholinesterase (BChE) is a highly sialylated, tetrameric serum protein, investigated as a bioscavenger for organophosphorous nerve agents. Expression of recombinant BChE (rBChE) in *Nicotiana benthamiana* results in accumulation of both monomers as well as assembled oligomers. In particular, we show here that co-expression of BChE with a novel gene-stacking vector, carrying six mammalian genes necessary for *in planta* protein sialylation, resulted in the generation of rBChE decorated with sialylated *N*-glycans. The *N*-glycosylation profile of monomeric rBChE secreted to the apoplast largely resembles the plasma-derived orthologue. In contrast, rBChE purified from total soluble protein extracts was decorated with a significant portion of ER-typical oligomannosidic structures. Biochemical analyses and live-cell imaging experiments indicated that impaired *N*-glycan processing is due to aberrant deposition of rBChE oligomers in the endoplasmic reticulum or endoplasmic-reticulum-derived compartments. In summary, we show the assembly of rBChE multimers, however, also points to the need for in-depth studies to explain the unexpected subcellular targeting of oligomeric BChE in plants.

## Introduction

Recombinant protein-based drugs are among the fastest growing branches of the pharmaceutical industry. Consequently, there is a demand for exploring new expression systems that allow faster, more flexible and possibly less expensive production than established platforms. In addition, new expression systems should preferably allow generation of biopharmaceuticals with enhanced bioactivities. For several reasons, plants have been considered a production system for therapeutic proteins (for recent review see Melnik and Stoger, 2013). Their glycosylation characteristics may well be the key to their success in commercial application. Recombinant glucocerebrosidase produced in carrot cells, and recently approved by the FDA, is an example of a therapeutic protein effectively produced in a plant-based expression system. Vacuolar targeting in these cells resulted in a largely homogeneous plant-typical glycosylated recombinant enzyme with enhanced *in vivo* activities compared with its mammalian cell-produced counterpart (Aviezer *et al.*, 2009; Shaaltiel *et al.*, 2007). The homogeneous *N*-glycosylation profile of plant-produced glucocerebrosidase clearly has advantages over commercially CHO-produced glucocerebrosidase, which requires extensive, and therefore expensive, glycan modelling procedures after harvesting or purification (Van Patten *et al.*, 2007).

Two unique features of plant glycosylation still limit their potential as a versatile expression platform: (i) plant-specific nonmammalian glycosylation and (ii) truncated glycosylation. With few exceptions (e.g. Lewis-a structures), plant complex *N*-glycans terminate with *N*-acetylglucosamine residues. However, in mammals, such structures are normally elongated by β1,4-galactosylation and in some cases by further sialylation. To address these issues, a series of knock-in and knock-out/down approaches were employed to humanize plant glycosylation (e.g. Bakker *et al.*, 2001; Cox *et al.*, 2006; Palacpac *et al.*, 1999; Strasser *et al.*, 2008; for recent reviews, see Bosch *et al.*, 2013; Castilho and Steinkellner, 2012). Notably, multisialylated *N*- and *O*-glycosylated proteins were generated, which required the introduction of an entire mammalian metabolic pathway into plants (Castilho *et al.*, 2010, 2012, 2013). Moreover, proteins produced in such glyco-modified plants exhibit enhanced *in vitro* and *in vivo* activities (Cox *et al.*, 2006; Loos and Steinkellner, 2012).

Sialylated structures, common on mammalian glycoproteins, are critical for the biological activity of many (therapeutic) proteins. However, this is a particularly difficult glycan modification to control in current mammalian-based expression systems (Hossler *et al.*, 2009; Taschwer *et al.*, 2012; Trummer *et al.*, 2006). Many recombinant proteins produced in CHO cells are undersialylated and as a consequence exhibit suboptimal pharmacokinetic (PK) properties. A prominent example is the enzyme human butyrylcholinesterase (BChE), which is a candidate bioscavenger of organophosphorous nerve agents for use in pre- and post-exposure treatment (Geyer *et al.*, 2010a,b). Human plasma BChE has investigational drug status with the US Food and Drug Administration [www.ClinicalTrials.gov, Identifiers: NCT00333528 and NCT00333515, see (Weber *et al.*, 2011a)]. Availability of plasma-derived BChE is limited, and thus, over the last decade, there have been numerous attempts to achieve recombinant expression of BChE either in cells (Saxena *et al.*, 1998) or transgenic organisms (Brandizzi and Barlowe, 2013; Geyer *et al.*, 2010a,b; Huang *et al.*, 2007; Li *et al.*, 2008). Despite the promise of such systems in providing the necessary quantities of the bioscavenger, the inferior PK properties of recombinant human BChE (rBChE) as compared with those of the plasma-derived counterpart remain a major hurdle (Geyer *et al.*, 2010b; Ilyushin *et al.*, 2013). The PK properties of rBChE are affected by both glycosylation and the size (serum BChE mainly circulates as tetramers) of the protein (Rosenberg *et al.*, 2010; Saxena *et al.*, 1998). Attempts to increase the mass of rBChE through extensive post-purification efforts, so as to prolong its stability in the bloodstream, have been remarkably successful (Biberoglu *et al.*, 2012; Geyer *et al.*, 2010b; Ilyushin *et al.*, 2013), but come with an unacceptably high price tag.

In this study, we explored the ability of *Nicotiana benthamiana* expression system to produce correctly assembled BChE that is decorated with glycans resembling its plasma-derived counterpart. A single multigene vector was generated that carries six genes involved in the mammalian sialylation pathway. This vector was transiently co-expressed with BChE cDNA in wild-type (WT) *N. benthamiana* and ΔXT/FT, a glycosylation mutant lacking plant-specific β1,2-xylose and core α1,3-fucose residues (Strasser *et al.*, 2008). *N*-glycosylation analyses of rBChE secreted to the apoplast revealed complex *N*-glycans that were efficiently sialylated. In contrast, rBChE that was affinity-purified from total soluble protein extracts carried, in addition to sialylated structures, significant amounts of oligomannosidic glycans. Subcellular localization studies using confocal laser-scanning microscopy revealed that rBChE tagged with a fluorescent protein accumulates in the ER and ER-derived vesicles. Biochemical characterization showed that correctly assembled oligomers and monomers were present in total soluble proteins, but only monomers were efficiently secreted into the intercellular space.

## Results

### Expression and purification of ^FLAG^BChE

**Figure 1:**
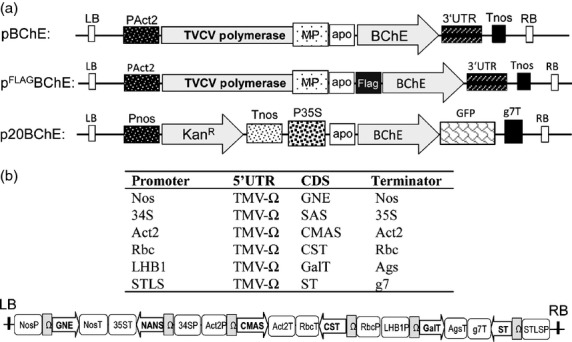
(a) Schematic representations of expression vectors. BChE cDNA was cloned into a modified TMV-based magnICON vector (pICHα26211) without (pBChE) and with an *N-*terminal FLAG-tag (p^FLAG^BChE). A fragment containing the barley alpha amylase signal peptide and BChE was cloned into a binary vector with a C-terminal GFP tag (p20BChE). Apo: Signal peptide sequence from barley α amylase; BChE: Human BChE sequence lacking the native signal peptide; FLAG: DYKDDDDK peptide sequence; GFP: green fluorescent protein; g7T: agrobacterium gene 7 terminator; Kan^R^: neomycin phosphotransferase 2 gene; LB: left border; MP: movement protein; PAct2: *Arabidopsis thaliana* actin 2 promoter; Pnos: nopaline synthase gene promoter; P35S: cauliflower mosaic virus 35S gene promoter; RB: right border; Tnos: nopaline synthase gene terminator; TVCV polymerase: turnip vein clearing virus RNA-dependent RNA polymerase; 3′UTR: TVCV 3′-untranslated region. (b) Schematic representation of the major features of the pICH88266 multigene vector. The figure displays the tandem cloning and relative orientation of the six expression cassettes. The basic elements used for the construction of the individual expression cassettes needed for protein sialylation are summarized in the table. LB: left border; NosP and NosT: nopaline synthase gene promoter and terminator; 34S P: cauliflower mosaic virus 34S gene promoter; 35ST: Cauliflower mosaic virus 35S gene terminator; Act2P and Act2T: *Arabidopsis thaliana* actin 2 promoter and terminator; RbcP and RbcT: *Arabidopsis thaliana* rubisco small unit 1 promoter and terminator; LHB1: *Arabidopsis thaliana* light-harvesting complex II chlorophyll a/b binding protein promoter; AgsT: agrocinopine synthase terminator; STLS: potato stem and leaf-specific promoter g7T, agrobacterium gene 7 terminator; TMV-Ω: tobacco mosaic virus 5′-untranslated region; RB: right border; GNE: UDP-*N-*acetylglucosamine 2-epimerase/*N-*acetylmannosamine-kinase; NANS: *N-*acetylneuraminic acid phosphate-synthase; CMAS: CMP-Neu5Ac synthetase; CST: CMP-Neu5Ac transporter; GalT: fusion of α2,6-sialyltransferase CTS region to the catalytic domain of human β1,4-galactosyltransfease (Strasser *et al.*, 2009); ST: α2,6-sialyltransferase.

**Figure 2:**
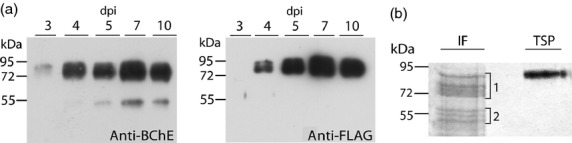
(a) Monitoring of ^FLAG^BChE expression in ΔXT/FT. Immunoblot analysis of total soluble proteins extracted from leaves infiltrated with p^FLAG^BChE using antibodies against the protein (anti-BChE, left panel) or the tag (anti-FLAG, right panel). dpi: days post infiltration. (b) Purification of ^FLAG^BChE_sia_. p^FLAG^BChE was co-infiltrated with pICH88266 multigene vector described in Figure 1. Coomassie Brilliant Blue-stained gel of ^FLAG^BChE_sia_ derived from IF (IF) and purified from TSP. Brackets 1 and 2 indicate ^FLAG^BChE_sia_ clusters.

A synthetic gene encoding plant-expression optimized human BChE (Geyer *et al.*, 2010a) was cloned into a TMV-based magnICON vector carrying a FLAG tag for purification (^FLAG^BChE Figure 1a). ^FLAG^BChE was delivered by agroinfiltration into leaves of *N. benthamiana* ΔXT/FT, a glycosylation mutant lacking plant-specific glycosylation (Strasser *et al.*, 2008). Expression of recombinant ^FLAG^BChE was monitored by Western blotting and revealed a specific signal between 72 and 95 kDa in accordance with the predicted size of the glycosylated monomer (85 kDa, Figure 2a). Highest expression was achieved at 7 days postinfiltration (dpi). After 5 dpi, a degradation product of approximately 55 kDa was detected in addition to the full-length protein. This product was particularly pronounced in samples extracted from the intercellular fluid (IF) representing the secreted fractions of the protein. FLAG-specific antibodies do not react with the degradation product, indicating that most probably the 55-kDa band is a result of N-terminal truncation of the protein. Human BChE is heavily glycosylated, and each monomer carries sialylated glycans on all of its nine *N*-glycosylation sites (Kolarich *et al.*, 2008). To ensure sialylation, ^FLAG^BChE was co-infiltrated with agrobacteria carrying a new multigene vector (pICH88266, see Experimental procedures) that contains all six genes necessary for *in planta* protein sialylation as recently described (Castilho *et al.*, 2008, 2010; Strasser *et al.*, 2009). The resulting product, ^FLAG^BChE_sia_, was collected from the IF and was resolved by SDS-PAGE to exhibit two broad clusters of bands, one between 72 and 95-kDa (Figure 2b, cluster 1), and another, referred to as ‘degraded rBChE’ below 55 kDa (Figure 2b, cluster 2), In contrast, ^FLAG^BChE_sia_ purified from TSP via FLAG-affinity chromatography revealed a single band corresponding to the largest band of IF-derived ^FLAG^BChE_sia_ kDa (Figure 2b). This band reacted with both BChE and FLAG-specific antibodies (Figure S1).

### *N*-Glycan analysis

To determine the exact *N*-glycosylation status of ^FLAG^BChE glycovariants, affinity-purified and IF-derived proteins were subjected to liquid chromatography–electrospray ionization mass spectrometry (LC-ESI-MS, Kolarich *et al.*, 2008; Pabst *et al.*, 2012; Stadlmann *et al.*, 2008). Essentially, all ^FLAG^BChE derived from the IF exhibited complex *N*-glycosylation. A single dominant *N*-glycoform was present, and depending on whether pICH88266 was co-expressed or not, it was, respectively, identified as GnGn and NaNa (Table 1). The *N*-glycosylation profile of purified ^FLAG^BChE and ^FLAG^BChE_sia_ differs from the IF-derived counterparts insofar as preparations of the former contained a significant fraction of oligomannosidic structures (50%–60%) in addition to complex structures (Table 1). Similar results were obtained when ^FLAG^BChE was expressed in *N. benthamiana* WT.

**Table 1:** Relative abundance in% of major glyco-structures detected on ^FLAG^BChE expressed in WT and ΔXT/FT plants. BChE was either collected from intercellular fluid (IF) or purified from total soluble proteins (TSP). ∑other ≤5%: sum of glyco-forms present at levels below 5%. See also Figure S2. The glycan structures are assigned using the ProGlycAn nomenclature (www.proglycan.com)

Structure	ΔXT/FT				WT	
	^FLAG^BChE (TSP)	^FLAG^BChE_sia_ (TSP)	^FLAG^BChE (IF)	^FLAG^BChE_sia_ (IF)	^FLAG^BChE_sia_ (TSP)	^FLAG^BChE_sia_ (IF)
MGn_iso_	8		6			
GnGn	27	6	86	9		6
Man 7	6	7				
Man 8	26	21			17	
Man 9	27	20			20	
GnGnXF					10	
MNaXF					15	
ANaXF					15	
NaNa		37		85		
NaNaXF					17	90
∑other ≤5%	6	9	8	6	6	4

Glycosite-specific analysis of TSP-purified ^FLAG^BChE_sia_ corroborated these findings (Figure S2). Tryptic digestion allowed us to identify five of the nine BChE glycosylation sites, namely Asn85, Asn269, Asn284, Asn396 and Asn483 corresponding to the tryptic glycopeptides (Gps) 2, 4, 5, 6 and 7. Our analysis could not resolve the adjacent glycosylation sites Asn513 and Asn514, which reside on the same tryptic peptide (Kolarich *et al.*, 2008), and we could not detect the tryptic glycopeptides Gp1 and Gp3, containing, respectively, the Asn45 and Asn134 glycosylation sites. The unexpected glyocosylation profile of ^FLAG^BChE and ^FLAG^BChE_sia_ purified from TSP suggests partial aberrant subcellular targeting.

### Subcellular localization of rBChE by confocal microscopy

**Figure 3:**
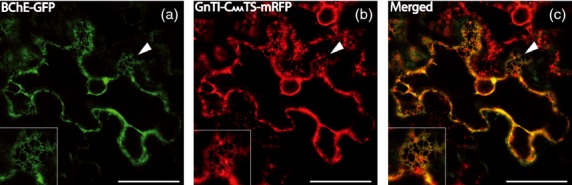
Subcellular localization of rBChE in *Nicotiana benthamiana* leaf epidermal cells. Expression of p20BChE (rBChE-GFP) and GnTI-C_AAA_TS-mRFP (a fusion protein that is predominantly endoplasmic-reticulum-retained) was monitored 2 dpi by live-cell confocal laser scanning microscopy. (a) CLSM image of a cell expressing p20BChE; (b) CLSM image of GnTI-C_AAA_TS-mRFP expressed in the same cell. Punctate structures represent Golgi bodies as frequently observed with GnTI-C_AAA_TS-mRFP (Farid *et al.*, 2011; Schoberer *et al.*, 2009); (c) corresponding overlay of both images. Co-localization appears in yellow. Boxed insets (bottom left corner) show a higher magnification of the region indicated by arrowheads. A significant co-localization of the two constructs was observed. Scale bar = 40 μm for all images.

A GFP-tagged BChE fusion protein was generated (rBChE-GFP, p20BChE; Figure 1a) to monitor the subcellular localization of recombinant BChE. The integrity of rBChE-GFP was confirmed by immunoblot using antibodies against GFP to exclude artefactual staining derived from free GFP (Figure S3). *Nicotiana benthamiana* leaf epidermal cells expressing rBChE-GFP were examined by live-cell confocal laser scanning microscopy (CLSM) at 2 dpi. A reticulate staining pattern typical for the cortical ER network was visible in many cells (Figure 3, panel A). Co-localization of rBChE-GFP with a predominantly ER-retained mRFP fusion protein (GnTI-C_AAA_TS-mRFP, Schoberer *et al.*, 2009) strongly suggested at least partial retention of rBChE-GFP in the ER (Figure 3). The results agree with the data obtained by *N*-glycosylation analyses of ^FLAG^BChE (and ^FLAG^BChE_sia_) purified from TSP (Figure S2).

### Eliciting factors that influence BChE targeting

**Figure 4:**
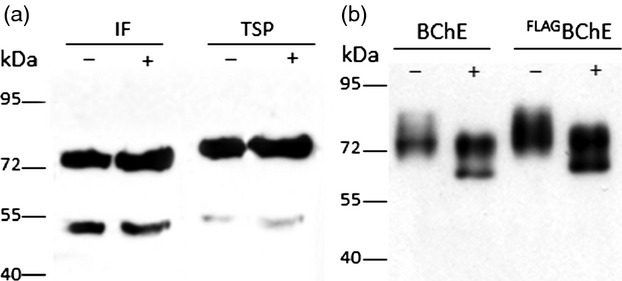
(a) Comparison of BChE expression in intercellular fluid (IF) and total soluble proteins (TSP) with (+) or without (−) a FLAG tag. The presence of the recombinant enzyme was monitored by Western blot analysis with anti-BChE antibodies. Both proteins were secreted to a similar extent. (b) Endo H treatment of rBChE to assess the presence of oligomannisidic *N*-glycans. TSP-containing BChE or ^FLAG^BChE was incubated with (+) or without (−) Endo H and subsequently subjected to immunoblot analysis using BChE-specific antibodies. The fraction of BChE sensitive to Endo H was essentially the same for both forms of the protein.

The presence of rBChE with and without FLAG tag was monitored side-by-side in IF and TSP to investigate whether the tag influences the secretion of rBChE. Western blotting exhibited a similar staining pattern for both tagged and nontagged variants, in IF and TSP, indicating that the FLAG tag does not significantly alter secretion (Figure 4a). In addition, an endoglycosidase H (Endo H) digestion was performed to roughly estimate the amount of oligomannosidic *N*-glycans in both versions (Endo H cleaves high mannose and some hybrid oligosaccharides from *N*-linked glycoproteins, but not complex *N*-glycans). Western blot analysis using BChE-specific antibodies showed that part of the immunoreactive band shifts to a smaller size upon Endo H treatment (Figure 4b). This fraction represents the part sensitive to Endo H. Glycoforms resistant to Endo H carry complex glycans (i.e. GnGn structures). Both tagged and nontagged BChE samples showed similar sensitivity to Endo H, indicating that there were no major differences in the glycosylation status of the two versions. This indirectly points to a similar extent of ER retention of tagged and nontagged rBChE in agreement with the above described *N*-glycan analyses and CLSM studies. Endo H treatment of IF-derived ^FLAG^BChE did not shift the protein band as expected as this fraction carries only complex *N*-glycan structures (Figure S4).

**Figure 5:**
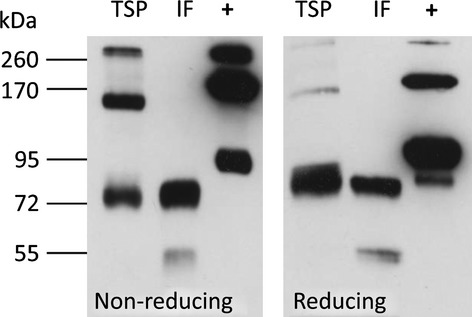
Analysis of the oligomerization status of ^FLAG^BChE. ^FLAG^BChE extracted from TSP and IF expressed in WT plants was subjected to immunoblotting under reducing and nonreducing conditions. Bands migrating at 75, 100 and 300 kDa represent monomers, dimers and tetramers, respectively. +: Human plasma BChE. Discrepancies between band sizes of plasma protein and ^FLAG^BChE are probably due to differences in *N*-glycosylation.

To determine the oligomerization status of ^FLAG^BChE, gel electrophoresis under nonreducing conditions followed by immunoblotting analysis was performed. The results showed the presence of monomeric, dimeric and tetrameric variants in TSP samples, however, only monomeric BChE was detected in the IF (Figure 5). The results point to inefficient secretion of oligomeric rBChE in *N. benthamiana*. It seems that dimers and tetramers are selectively retained in the ER or deposited in ER-derived vesicles.

## Discussion

Here, we report a novel multigene vector that enables the reconstitution of the entire biosynthetic pathway for protein sialylation in plants. rBChE efficiently decorated with sialylated *N*-glycans was produced by co-infiltration with only two recombinant agrobacterial strains (one for the glycan modelling and one for the expression of the gene of interest). Notably, the targeted glycosylation profile of rBChE secreted to the apoplast largely resembles that of the plasma-derived counterpart, an important prerequisite for a highly biologically active molecule in humans. Expression platforms that allow control over protein sialylation are required for the production of most protein-based drugs, which need to be stable in the bloodstream. So far, such control is largely lacking or ineffective in mammalian-based expression systems. Proof of concept studies for *in planta* protein sialylation has recently been reported (Castilho *et al.*, 2010). The procedure required the simultaneous delivery of six agrobacterial strains, each carrying a binary vector with cDNA encoding the respective mammalian glycosylation enzyme. This approach may lead to glycosylation inconsistencies, because all genes must work in a highly coordinated fashion at a single cell level, and this problem is exacerbated when large-scale production is considered. Our solution was to simplify the procedure by reducing the number of binary vectors that need to be co-delivered into plants. In this study, we achieved *in planta* protein sialylation by the coordinated action of all glycosylation proteins delivered by a single multigene vector. This is remarkable, considering the complexity of the procedure, and provides a viable alternative to transgenic methods. Different glycoforms can be straightforwardly generated by changing the composition of the multigene vector. We have found that medium-scale infiltrations with various reporter genes result in largely homogeneous glycosylation profiles (A. Castilho, unpublished results). Importantly, the use of multigene vectors did not obviously alter expression levels of rBChE as determined by Western blot analysis. To reduce the risk of transgene silencing, the expression cassettes on the multigene vector employ several promoter–terminator combinations. Our approach can be generally applied to any recombinant protein, and it can potentially be transferred to other plants species.

Various plant species are currently under investigation as production platforms because the optimum accumulation of different proteins depends on the characteristics of the protein and the specifics of the plant platform. These include dicots (e.g. *Nicotiana* species, Both *et al.*, 2013), monocots (e.g. cereals, Rademacher *et al.*, 2008) and cells (e.g. Schillberg *et al.*, 2013). However, not all relevant glyco-traits are present in all of these plant species. The near future could see humanization of glycosylation by the use of multigene vectors transiently expressed in a plant species of preference. On the other hand, confinement to a limited number of commercially exploited plant expression systems is also to be expected (Klimyuk *et al.*, 2012; Wolfson, 2013). We open the door in both directions with the procedure described here. Moreover, our results may encourage scientists in other fields to introduce other complex traits into plants, such as tolerance to biotic/abiotic stress or improvement of food and feed quality (Bock, 2013).

We show that *N. benthamiana*-derived BChE can form dimers and tetramers in agreement with previous results (Geyer *et al.*, 2010b), and our results exhibit a high degree of conservation between the secretory pathways of plants and mammals. Recombinant BChE monomers are efficiently secreted to the apoplast, following the typical secretory pathway from the ER through the Golgi. ER-associated deposition of rBChE oligomers and subsequent oligomannosidic glycosylation came as a surprise because the enzyme was designed for secretion. It seems that the stringent control systems that operate at the ER–Golgi interface (Brandizzi and Barlowe, 2013) prevent the release of oligomeric rBChE from the ER. In mammalian cells, large molecules are transported via vesicles termed COP II (Miller and Schekman, 2013). Plant equivalents have so far not been identified. Factors that influence intracellular transport of proteins in plants are still poorly characterized (Denecke *et al.*, 2012). Previous studies have shown that inadvertent retention of protein in the ER can be due to improper folding or stress-induced alterations in ER conditions (Liu and Howell, 2010). Agrobacterial-induced alteration of the secretory pathway also cannot be excluded, albeit recent studies using a transgenic approach did show unexpected localization of recombinant proteins in ER-derived vesicles as well (Loos *et al.*, 2011a,b). In most eukaryotic cells, assembly and secretion of proteins are largely controlled by ER-resident chaperons. This is particularly well demonstrated for immunoglobulins (Feige *et al.*, 2010). Secretion of correctly assembled IgG heterodimers is associated with binding to the evolutionarily well-conserved chaperone BiP. IgG variants that lack certain BiP-binding domains are not correctly folded and retained in the ER (Feige *et al.*, 2009). Association of plant-produced antibodies with BiP was previously reported (Nuttall *et al.*, 2002), suggesting similar molecular control mechanisms in plant and mammalian cells. Factors that influence BChE secretion remain elusive, so far. However, targeted subcellular localization of (recombinant) proteins should be feasible as more is understood about signals that drive this fundamental biological process in cells.

The results presented here are encouraging for generating rBChE (and other plasma circulating proteins) suitable for therapeutic application with a PK profile equivalent to that of the human-plasma-derived counterpart. Together with recent results obtained by *in vivo* studies of plant-derived rBChE (Geyer *et al.*, 2010a,b), it appears entirely feasible that this proof of concept study will translate into a pharmaceutically valuable product. However, we also point to the necessity to further elucidate factors that are involved in subcellular targeting of (recombinant) proteins.

## Experimental procedures

### MagnICON vectors for the expression of BChE in *Nicotiana benthamiana*

To target proteins to the secretory pathway, the signal peptide from the barley α-amylase gene was inserted into the tobacco mosaic virus (TMV)-based magnICON vector pICH26211 (Marillonnet *et al.*, 2004, 2005), resulting in the vector pICHα26211. Additionally, a FLAG peptide (DYKDDDDKGG) was inserted into pICHα26211 for *N-*terminal tagging of proteins generating pICHα^FLAG^26211. Construction of pICH26211 (courtesy of Icon Genetics, GmbH), based on pICH18711, was previously described (Marillonnet *et al.*, 2005). Human BChE cDNA, codon optimized for dicotyledonous plants (GeneArt, Regensburg, Germany), was used as a template for PCR amplification using primers with flanking *BsmB*I restriction sites (BChE-F1: 5′-GCACGTCTCAAGGTGAGGATGACATCATCATTGC-3′; BChE-R1: 5′-GCACGTCTCAAAGCCTAGAGACCCACACAGCTCTCC-3′); the resulting fragment corresponds to full-length butyrylcholinesterase without its signal peptide. Subsequently, the PCR fragment was inserted into the Zero Blunt® TOPO® vector (Invitrogen®, Vienna, Austria), and DNA sequence analysis confirmed its identity. After digestion with *BsmB*I, the DNA fragment was cloned into the *Bsa*I sites of the pICHα26211 and pICHα^FLAG^26211 vectors. The resulting vectors pBChE and p^FLAG^BChE (Figure 1a) were introduced into the *Agrobacterium tumefaciens* strain GV3101 pMP90 by electroporation.

### Multigene vector for modulation of ^FLAG^BChE N-glycosylation

The multigene vector pICH88266 consists of six expression cassettes each carrying one of the genes required for synthesis and transfer of sialic acid to *N*-glycans. Assembly of the different expression cassettes in a single construct was performed using the Golden Gate cloning technology (Engler and Marillonnet, 2013) in conjunction with the modular cloning system for multigene constructs (Weber *et al.*, 2011b; Werner *et al.*, 2012). Each expression cassette consists of a promoter, a 5′ untranslated region (5′UTR), the target protein coding sequence (CDS) and a terminator. The expression of each gene was initially tested under different combinations of promoters and terminators, and Figure 1b shows a schematic representation of the multigene vector and lists the selected features for all six expression cassettes. The features of the mammalian genes for *in planta* sialylation are as described in Castilho *et al.*, 2008, 2010 and Strasser *et al.*, 2009. The modular cloning system is designed in such a way that one can assemble six expression cassettes in one reaction. The multigene vector was transformed into agrobacterium strain UIA 143 pMP90.

### Plant material and protein expression

*Nicotiana benthamiana* wild-type (WT) and mutant plants, which are devoid of plant-specific β1,2-xylose and core α1,3-fucose residues (ΔXT/FT) (Strasser *et al.*, 2008), were cultivated in a growth chamber with a constant temperature of 24 °C, 60% relative humidity and a 16 h light/8 h dark photoperiod. For agroinfiltration experiments, 4- to 5-week-old plants were used. Agrobacteria containing BChE were infiltrated at an optical density at 600 nm (OD_600_) of 0.1, whereas the OD_600_ of pICH88266 was 0.05.

### Isolation of total soluble protein

Total soluble proteins (TSPs) were extracted from 250 mg of infiltrated leaf material. The leaf material was ground with steel beads in a swing mill (Retsch®, MM2000, Haan, Germany) for 2 min at amplitude 60 and then double volume (v/w) of 1×PBS was added. The extracts were incubated on ice for 10 min and centrifuged (3500 ***g***, 5 min at 4 °C). Total soluble protein was quantified using the Bradford-based BioRad® Protein Assay (Biorad, Munich, Germany) following the manufacturer's instructions.

### Purification of rBChE

BChE and ^FLAG^BChE were either partially purified by collection of proteins secreted into the apoplast (intercellular fluid, IF) (Castilho *et al.*, 2011) or for ^FLAG^BChE the protein was also purified from TSP by affinity chromatography with Anti-FLAG® M2 Affinity Gel (1 mL; Sigma Aldrich Handels Gmbh®, Vienna, Austria) according to the manufacturer's instructions. Elution from the gel was performed with FLAG peptide (50 mm TBS containing 100 μg/mL FLAG® peptide; Sigma Aldrich®). Eluates were analysed by Western blotting. Fractions that showed strong signals for ^FLAG^BChE were pooled and dialysed overnight at 4 °C against 1×PBS containing 0.02% sodium azide. The sample was then concentrated sixfold with a 10-kDa cut-off Amicon® Ultra-4 Centrifugal Filter Unit (Millipore GmbH®, Vienna, Austria) before loading onto a gel.

### Immunoblot analysis of rBChE

Prior to separation by 10% SDS-PAGE, TSP- and IF-derived proteins were mixed with reducing 4× Laemmli buffer and incubated for 5 min at 95 °C. In experiments where nonreducing conditions were required, β-mercaptoethanol was excluded from 4× Laemmli buffer. Fractionated proteins were used for immunoblotting or Coomassie Brilliant Blue R-250 staining. Protein- and tag-specific antibodies were used for immunoblot analysis (polyclonal goat anti-BChE N-15; Santa Cruz Biotechnology, Inc® (Heidelberg, Germany) sc-46803, diluted 1 : 300 and monoclonal mouse anti-FLAG M2, F3165; Sigma Aldrich®, diluted 1 : 10000). Detection was performed using HRP-conjugated secondary antibodies (anti-goat IgG-peroxidase antibody A5420, and anti-mouse IgG-peroxidase antibody A9044, both diluted 1 : 10000 from Sigma Aldrich®). Clarity™ Western ECL from Bio-Rad was used as a substrate.

### Glycoprofiling of ^FLAG^BChE

Oligosaccharides of ^FLAG^BChE (total glycans) were analysed by LC-ESI-MS after treatment of the samples with PNGase A (Castilho *et al.*, 2010; Kolarich *et al.*, 2008). Quantitation was based on areas covered by the elution profiles as well as signal heights of the deconvoluted spectra. Glycopeptides of ^FLAG^BChE were analysed by LC-ESI-MS as described previously (Pabst *et al.*, 2012; Stadlmann *et al.*, 2008). In brief, the samples were submitted to denaturing SDS-PAGE, and the immunoreactive bands were S-alkylated, digested with trypsin and extracted from the gel fragment with 50% acetonitrile. Samples were separated on a Biobasic C18 column (150 × 0.32 mm; Thermo Electron, Niederelbert, Germany) with a gradient of 1%–60% acetonitrile containing 65 mm ammonium formate at pH 3.0. Positive ions were detected with a quadrupole time of flight (Q-TOF) Ultima Global mass spectrometer (Waters, Milford, MA). Glycoforms were quantified by the summed and deconvoluted spectra of the glycopeptides' elution profile. Peaks were labelled according to the ProGlycAn system (www.proglycan.com). Trypsin digestion allowed the analysis of five of the nine BChE glycopeptides (Gps): Gp2: W^80^SDIWNATK^88^; Gp4: N^269^RTLNLAK^276^; Gp5: E^283^NETEIIK^290^; Gp6: E^368^NNSIITRK^376^ and Gp7: D^482^NYTKAEEILSR^493^. Glycosites 8 and 9 (Y^505^GNPNETQNNSTSWPVFK^522^) are part of the same tryptic peptide. Therefore, the discrimination of the glycosylation on the distinct glycosites is arduous, as already observed for hBChE in previous studies (Kolarich *et al.*, 2008).

### Subcellular localization by confocal laser scanning microscopy

BChE was *C-*terminally tagged with green fluorescent protein (GFP) for subcellular localization experiments. A fragment containing the signal peptide of the barley alpha amylase and the BChE-coding sequence lacking the stop codon was PCR amplified from pBChE with the primers amylase F2 (5′-TATAACTAGTATGGCAAACAAACACCTCTCACT-3′) and BChE-R2 (5′-tataactagtgagacccacacagctctccttc-3′), digested with *Spe*I and cloned into the *Xba*I site of the binary plant expression vector p20 (Castilho *et al.*, 2010). The resulting fusion construct p20BChE (Figure 1a) was transformed into the *Agrobacterium tumefaciens* strain UIA 143 pMP90. p20BChE was transiently expressed in *N. benthamiana* leaf epidermal cells using agrobacterium-mediated infiltration at an OD_600_ of 0.2. GnTI-C_AAA_TS-mRFP (Schoberer *et al.*, 2009) was co-infiltrated with p20BChE at an OD_600_ of 0.03 and used as an ER marker for co-localization studies. Monomeric red fluorescent protein (mRFP) and GFP expression were monitored at two dpi using an upright Leica TCS SP5 confocal laser scanning microscope. Dual-colour imaging of GFP- and mRFP-expressing cells was performed simultaneously using a 488-nm argon laser line and a 561-nm helium/neon laser line. Post-acquisition image processing was performed in Adobe Photoshop CS4.

### Endo H treatment

Total soluble protein and IF samples of BChE and ^FLAG^BChE were boiled in buffer containing 0.5% SDS and 0.04 m DTT for 10 min. Then, the samples were either mock-digested or digested in the presence of 1000 units of endoglycosidase H (New England Biolabs, Beverly, MA) in 50 mm sodium citrate, pH 5.5, for 1 h at 37 °C. The samples were then analysed by reducing 10% SDS-PAGE and Western blot analysis with anti-BChE antibodies.

## Supporting Information

Figure S1 Purification of sialylated FLAG-tagged BChE (^FLAG^BChE_sia_). **Figure S2** Site-specific *N-*Glycan profiles of ^FLAG^BChE_sia_. **Figure S3** Western blot analysis of GFP constructs used for confocal laser scanning microscopy. **Figure S4** Endo H treatment of IF-derived rBChE.
