# Supplementary material for: Oligomerization status influences subcellular deposition and glycosylation of recombinant butyrylcholinesterase in Nicotiana benthamiana
Source: Plant Biotechnol J. 2014 Mar 11;12(7):832–9. doi: 10.1111/pbi.12184 (PMC4265266; doi:10.1111/pbi.12184)
Supplement: Figure S1 — Purification of sialylated FLAG-tagged BChE (FLAGBChEsia). Figure S2 Site-specific N-Glycan profiles of FLAGBChEsia. Figure S3 Western blot analysis of GFP constructs used for confocal laser scanning microscopy. Figure S4 Endo H treatment of IF-derived rBChE. [file pbi0012-0832-SD1.pdf]

Supplemental Data

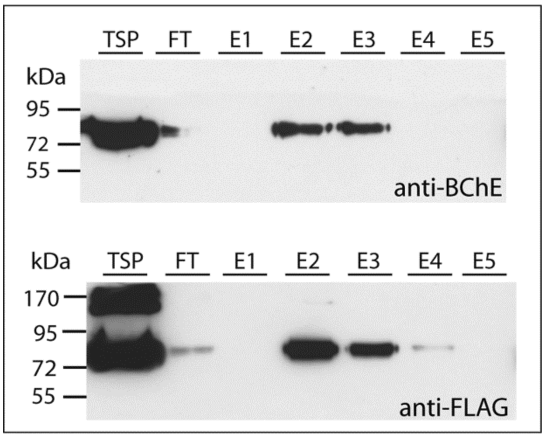

Figure S1. Purification of sialylated FLAG-tagged BChE ($^{FLAG}BChE_{sia}$). Western blot analysis of different stages of the purification procedure using antibodies against BChE (top panel) and FLAG-tag (bottom panel). Total soluble protein (TSP, starting material), flow through (FT) and eluates 1 to 5 (E1-5) were applied to the gel. In addition, FLAG-specific antibodies detected a signal at 170 kDa in TSP (bottom panel), which is most likely due to an unspecific binding of the FLAG antibody since it does not react to BChE-specific antibodies (top panel). The molecular weights are shown in kilodalton (kDa).

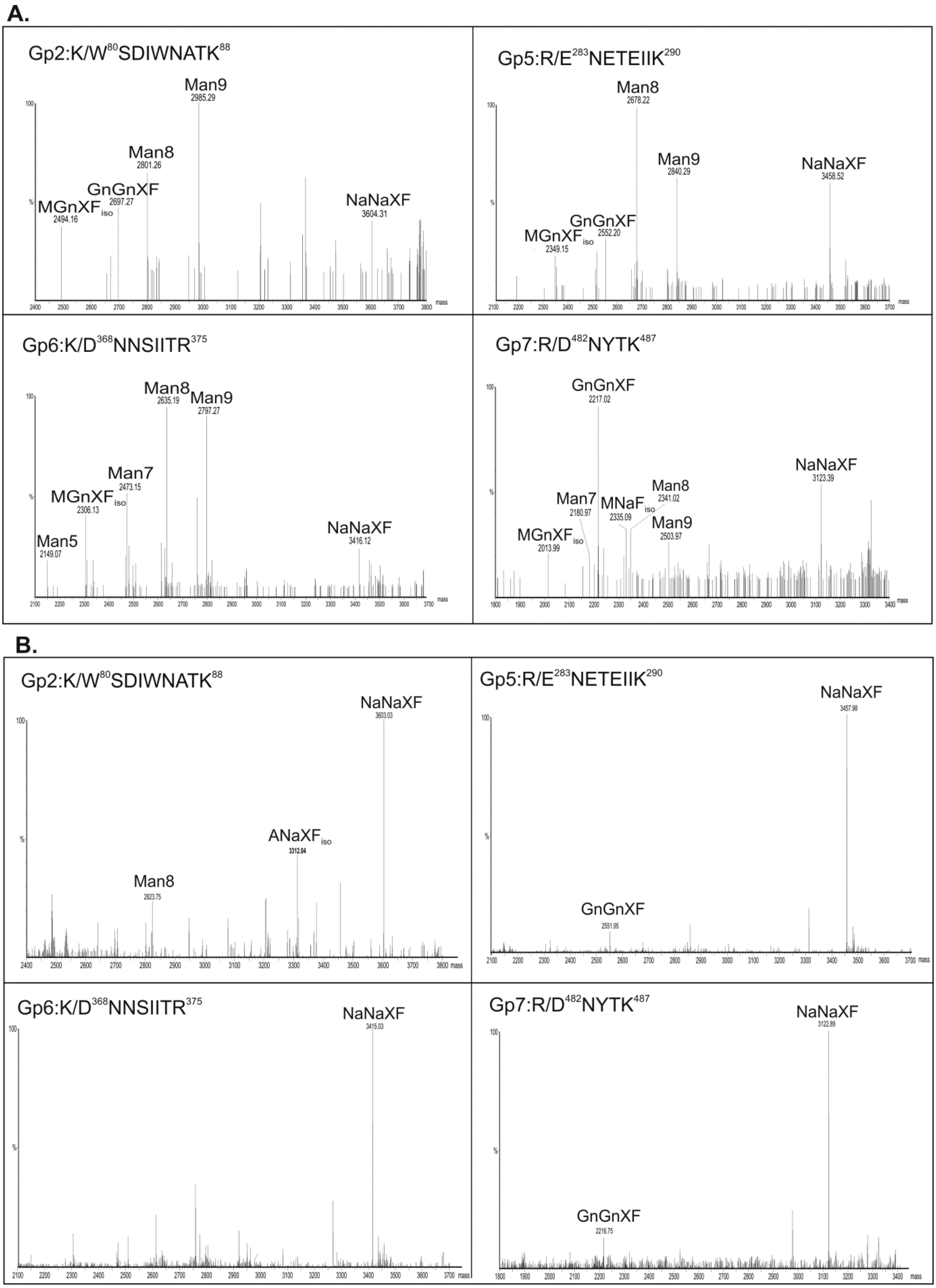

Figure S2. Site specific *N*-Glycan profiles of ^{FLAG}BChE$_{sia}$. ^{FLAG}rBChE was co-expressed transiently in *N. benthamiana* wild-type plants with the multigene vector for the expression of genes necessary for *in planta* sialylation. ^{FLAG}BChE$_{sia}$ was either purified from TSP (A) or collected from the intercellular fluid (B). *N*-glycosylation profiles of glycopeptides (Gp) 2, 5, 6, and 7 are shown. Peaks

were labeled in accordance with the ProGlycAn system (www.proglycan.com). The suffix "iso" at the end of glycan abbreviations denotes the probable presence of isomers. Unassigned peaks are background originating from co-eluting peptides of other proteins.

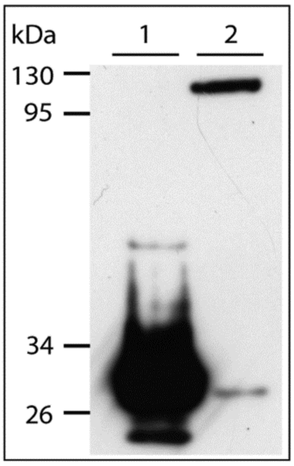

Figure S3. Western blot analysis of GFP constructs used for confocal laser scanning microscopy. TSP extracts from leaves infiltrated with p20 (free GFP, lane 1) and p20BChE (BChE-GFP, lane 2) were analyzed. Proteins were detected with anti-GFP antibodies (1:2000, Amsbio TP401). The 130-kDa band represents the size of the rBChE-GFP fusion protein, 26 kDa is the size of GFP. The molecular weights are shown in kilodalton (kDa).

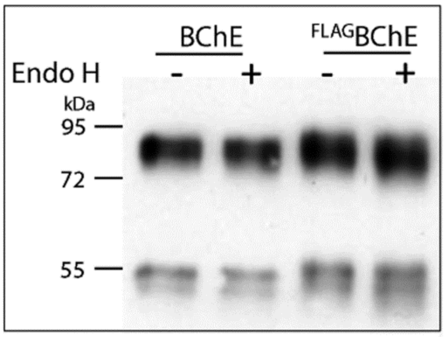

Figure S4. Endo H treatment of IF-derived rBChE. IF-extracted BChE (lanes 1 and 2) and ^{FLAG}BChE (lanes 3 and 4) were treated without (-) or with (+) Endo H and subsequently subjected to Western blot analysis using BChE-specific antibodies. The molecular weights are shown in kilodalton (kDa).
